# Analysis of Cutaneous Lupus Hospitalizations: A United States National Population-Based Study

**DOI:** 10.7759/cureus.38982

**Published:** 2023-05-13

**Authors:** Emily He, Christopher Hino, Osaigbokan Aihie, Anthonia Ijeli, Amaka C Ugoh, Anum Akhlaq, Olive C Osuoji, John Eboma, Joan Ezomo, Precious Onobraigho, Precious O Eseaton, Ehizogie Edigin

**Affiliations:** 1 Internal Medicine, Loma Linda University Medical Center, Loma Linda, USA; 2 School of Medicine, University of Missouri School of Medicine, Columbia, USA; 3 Internal Medicine, Springfield Clinic, Lincoln, USA; 4 Internal Medicine, University of Benin Teaching Hospital, Benin City, NGA; 5 Medicine, University of Mississippi Medical Center, Jackson, USA; 6 Dermatology Clinical Research, University of California San Diego, San Diego, USA; 7 Internal Medicine, University of Benin/Kazaure General Hospital, Kazaure, NGA; 8 Gastroenterology, Spire Manchester Hospital, Manchester, GBR; 9 Internal Medicine, University of Benin College of Medicine, Benin City, NGA; 10 Internal Medicine, John H. Stroger, Jr. Hospital of Cook County, Chicago, USA

**Keywords:** trend analysis, rheumatology, hospital resource utilization, inpatient mortality, systemic lupus erythematous, cutaneous lupus erythematous, national population-based study

## Abstract

Background

There are limited studies analyzing cutaneous lupus erythematosus (CLE) hospitalizations. In this study, we aimed to analyze baseline demographics of systemic lupus erythematosus (SLE) and CLE patients, identify the most common reasons for hospitalizations, and find out the hospitalization outcomes.

Materials and methods

We performed the analysis using the National (Nationwide) Inpatient Sample (NIS) database between 2016 and 2019. For the CLE cohort, data for adults aged 18 years and older with the primary or secondary diagnosis of CLE using International Classification of Disease - 10th revision (ICD-10) codes were extracted. For comparison, the SLE cohort was identified by patients aged 18 years and older with primary or secondary diagnoses of SLE using ICD-10 codes. Chi-squared test was used to compare baseline demographic characteristics. Multivariable logistic and linear regression was used to calculate outcomes of interest.

Results

In comparison to the SLE cohort, the CLE cohort was not only older in age and lower percentage female, but also had shorter length of stay, less total hospital charge, and the majority had Medicare as primary insurance. The SLE cohort included predominantly African American patients while the CLE cohort was majority Caucasian patients. The cardiovascular risks were more prevalent in the CLE cohort and most commonly admitted for sepsis, cardiovascular disease, and mental health disorders.

Conclusion

Our study highlights the importance of outpatient follow-up in CLE patients to closely monitor cardiovascular risk factors, early identification of infections, and routine mental health screenings to reduce hospitalizations and resource utilization.

## Introduction

Lupus can have a systemic form known as systemic lupus erythematosus (SLE) and a cutaneous form known as cutaneous lupus erythematosus (CLE). CLE is an autoimmune disease that primarily affects the skin. The dermatologic presentations can be divided into acute, subacute, and chronic subcategories [1,2]. In acute CLE, patients typically present with a malar rash [3]. Patients may also have generalized macules on sun-exposed skin [1]. In subacute CLE, patients can present with scaly erythematous plaques that may coalesce on sun-exposed skin [3-6]. Chronic CLE, or discoid lupus, presents with disk-shaped plaques, typically involving the head and neck. Discoid plaques can lead to permanent discoloration and scarring [4]. When these well-demarcated plaques occur on the scalp, they can cause scarring alopecia [4]. At times, these discoid plaques can occur on other body parts aside from the head and neck, and they typically are not painful and non-pruritic [4]. As CLE patients have photosensitive skin, sun protection is a cornerstone of prevention and progression of rashes [3,6].

Though CLE has been regarded as less severe and better prognosis than SLE, it can still impact a patient's quality of life and ability to work [1,7]. This not only causes direct financial loss to the patient but also indirect costs for the community from lack of workforce participation [7]. Also, previous studies have shown that CLE can progress to SLE [8,9]. Therefore, studies on CLE hospitalizations are important because they underline the economic and medical impact of CLE. 

While there has been a considerable amount of data on SLE hospitalization, large national population studies are limited on CLE hospitalizations. In this study, we aimed not only to analyze the most common reasons for hospitalization among CLE patients but also to compare baseline demographic characteristics and hospital outcomes in CLE patients to their SLE counterparts from 2016-2019.

This article was previously presented as a meeting abstract at the 2022 American Academy of Rheumatology (ACR) Convergence on November 14, 2022, in Philadelphia, United States.

## Materials and methods

Data source 

The National (Nationwide) Inpatient Sample (NIS) is the largest inpatient public database in the United States. The NIS is a healthcare cost and utilization project database that was created and maintained by the Agency for Healthcare Research and Quality. The NIS is a stratified sample of United States community hospitals. Hospitalizations are weighted to be nationally representative of the entire United States population. We obtained data from the NIS 2016-2019 databases. Each hospitalization in the NIS has a principal diagnosis (the main reason for hospitalization) and can have up to 39 secondary diagnoses. 

Study population 

For the CLE cohort, we extracted data for adult patients aged ≥18 years with either a principal or secondary diagnosis of CLE, using the ICD-10 code L93. CLE cohort included patients with discoid lupus erythematous, subacute CLE, lupus erythematous profundus, and lupus panniculitis. We excluded patients with any form of SLE using ICD-10 code M32 from the CLE cohort. For comparison, we obtained the SLE cohort by searching for patients aged ≥18 years with either a principal or secondary diagnosis of SLE with organ or system involvement using ICD-10 code “M321”. 

Statistical analysis 

Analyses were performed using Stata Statistical Software: Release 16 (2019; StataCorp LLC, College Station, Texas, United States). By using a “rank” command in Stata, the most common principal discharge diagnoses were divided into categories based on organ system, and the most common specific principal discharge diagnoses were recorded for the CLE cohort in descending order of frequency. We used chi-square test to compare baseline demographic characteristics between the CLE and SLE cohorts. We used multivariable logistic and linear regression analysis, adjusting for sex, age, Charlson comorbidity index (CCI), insurance status, and income for a categorical outcome (inpatient mortality) and continuous outcome (hospital length of stay and total hospital charges) between both cohorts. 

Institutional board review (IRB)

Since NIS contains publicly available depersonalized and de-identified patient data, IRB approval was waived. 

## Results

Approximately 142 million hospitalizations were included in the combined NIS databases between 2016 to 2019. Of these, the SLE cohort contained 130,475 hospitalizations (0.09%), while the CLE cohort contained 62,435 hospitalizations (0.04%). The CLE cohort was older in age, lower percentage female, lower inpatient mortality, shorter LOS, less total hospital charges, lower CCI score, fewer in the lowest household income quartile, and more Medicare insured compared to the SLE cohort (Table 1 and Table 2). 

**Table 1:** Baseline characteristics of patients hospitalized with CLE and SLE CLE: cutaneous lupus erythematosus; SLE: systemic lupus erythematosus; MI: myocardial infarction; PCI: percutaneous coronary intervention; CABG: coronary artery bypass graft; COPD: chronic obstructive pulmonary disease; DM: diabetes mellitus; CHF: chronic congestive heart failure; CKD: chronic kidney disease; O2: oxygen; A. Fib: atrial fibrillation Median household income means median household income for the patient’s Zone Improvement Plan (ZIP) code; Co-morbidities mean secondary diagnosis present at admission or developed during hospitalization

Baseline Characteristics	SLE hospitalizations (n=130475)	CLE hospitalizations (n=62435)	p-value
Age (mean)	42.3	58.5	<0.0001
Female, n (%)	109990 (84.3)	51384 (82.3)	<0.0001
Race, n (%)			<0.0001
White	33924 (26)	38959 (62.4)	
Black	58975 (45.2)	15421 (24.7)	
Hispanic	25704 (19.7)	5307 (8.5)	
Asian American	6393 (4.9)	937 (1.5)	
Native American	913 (0.7)	375 (0.6)	
Charlson comorbidity index, n (%)			<0.0001
0-2	37577 (28.8)	42830 (68.6)	
≥3	92898 (71.2)	19605 (31.4)	
Teaching hospital, n (%)	108686 (83.3)	41519 (66.5)	<0.0001
Urban hospital, n (%)	126430 (96.9)	55817 (89.4)	<0.0001
Expected primary payer, n (%)			<0.0001
Medicare	55713 (42.7)	34090 (54.6)	
Medicaid	35098 (26.9)	10114 (16.2)	
Private	34706 (26.6)	16483 (26.4)	
Self-pay	4958 (3.8)	1748 (2.8)	
Median household income (quartile), n (%)			<0.0001
1^st ^(0-25^th^)	49450 (37.9)	20604 (33)	
2^nd ^(26^th^-50^th^)	32097 (24.6)	16483 (26.4)	
3^rd^ (51^st^-75^th^)	28183 (21.6)	14735 (23.6)	
4^th^ (76^th-^100^th^)	20876 (16)	10614 (17)	
Co-morbidities, n (%)			
Dyslipidemia	27791 (21.3)	19480 (31.2)	<0.0001
Prior MI	5741 (4.4)	3933 (6.3)	<0.0001
A fib	8089 (6.2)	5931 (9.5)	<0.0001
COPD	9133 (7)	12112 (19.4)	<0.0001
Old stroke	10438 (8)	4995 (8)	0.9512
Hypertension	25312 (19.4)	24599 (39.4)	<0.0001
PVD	2218 (1.7)	2248 (3.6)	<0.0001
Hypothyroidism	15787 (12.1)	11488 (18.4)	<0.0001
DM type 1	783 (0.6)	437 (0.7)	0.2049
DM type 2	17223 (13.2)	12924 (20.7)	<0.0001
Obesity	17353 (13.3)	10364 (16.6)	<0.0001
CHF	30270 (23.2)	10676 (17.1)	<0.0001
CKD	79068 (60.6)	10864 (17.4)	<0.0001
Liver disease	6785 (5.2)	3621 (5.8)	0.0179
Maintenance hemodialysis	36142 (27.7)	1811 (2.9)	<0.0001
Smoking	14091 (10.8)	13798 (22.1)	<0.0001
Anemia	85983 (65.9)	19792 (31.7)	<0.0001

**Table 2:** Hospital outcomes of hospitalizations of patients with CLE and SLE CLE: cutaneous lupus erythematosus; SLE:  systemic lupus erythematosus; aOR: adjusted odds ratio; CI: confidence interval; USD: United States dollar

Hospital outcomes	SLE hospitalizations (n=130475)	CLE hospitalizations (n=62435)	aOR (95% CI),	p-value
Mortality, n %	3001 (2.3)	1124 (1.8)	0.67 (0.55-0.81)	<0.0001
LOS, days	6.8	4.7	-1.6 (1.8-1.4)	<0.0001
Total hospital charges, USD	83021	53200	-18927 (22177-15676)	<0.0001

**Figure 1:**
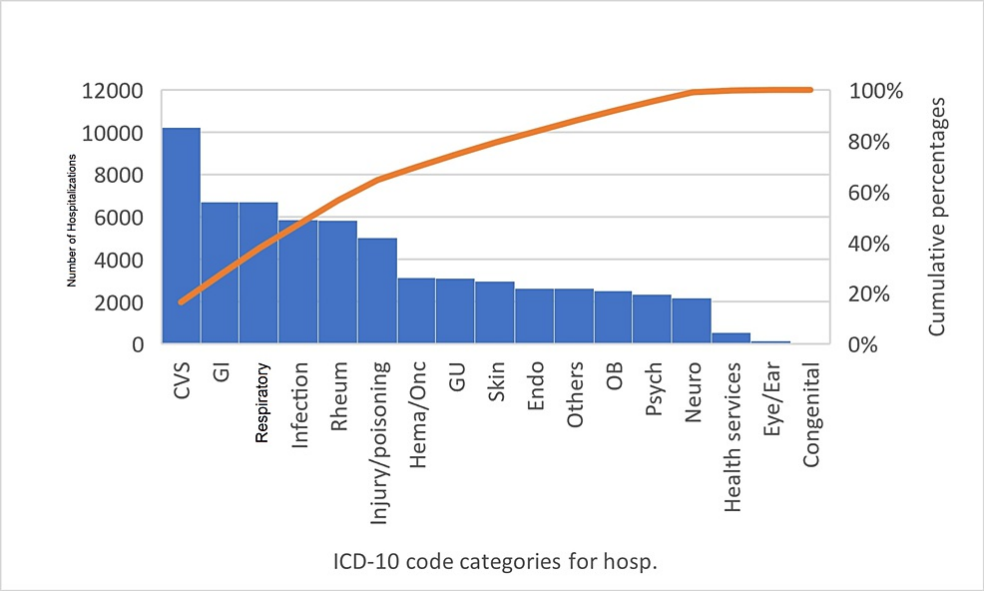
Pareto chart of ICD-10 code categories in descending order of frequency for hospitalization of patients with CLE. Line on the secondary axis plots the cumulative percentages of each ICD-10 code categories. Hema/Onc: neoplasms and diseases of the blood and blood-forming organs and certain disorders involving the immune mechanism; Endo: endocrine, nutritional, and metabolic diseases; Psych: mental, behavioral, and neurodevelopmental disorders; Neuro: diseases of the nervous system; CVS: cardiovascular system (diseases of the circulatory system); GI: gastrointestinal; Rheum: diseases of the musculoskeletal system and connective tissue, GU: genitourinary; ICD-10: International Classification of Diseases, 10th revision; CLE: cutaneous lupus erythematosus; Hosp: hospitalization Others include symptoms, signs, and abnormal clinical laboratory findings, not elsewhere classified; Health services include factors influencing health status and contact with health services

The CLE cohort had a greater percentage of White patients and fewer Black, Hispanic, Asian American, and Native American patients. Furthermore, fewer CLE patients presented to teaching or metropolitan hospitals compared to the SLE cohort. Although the SLE cohort had a higher incidence of congestive heart failure, chronic kidney disease, anemia, and need for maintenance dialysis, the CLE cohort had a greater percentage of cardiovascular (CV) risk factors such as dyslipidemia, prior myocardial infarction, hypertension, peripheral vascular disease, type 2 diabetes, obesity, and smoking. The most common principal diagnosis categories of hospitalizations of patients with CLE in descending order of frequency were CV, gastrointestinal, respiratory, infections, and rheumatologic disorders (Figure 1). 

The most common specific principal diagnoses for these patients in descending order of frequency were sepsis, acute chronic obstructive pulmonary disease exacerbation, pneumonia, acute kidney injury, and discoid lupus erythematous (Table 3). 

**Table 3:** ICD-10 code categories and specific principal discharge diagnosis for cutaneous lupus erythematosus hospitalizations* * Hospitalizations for patients with principal or secondary diagnosis of cutaneous lupus erythematosus. COPD: chronic obstructive pulmonary disease; CKD: chronic kidney disease; HF: heart failure; ICD-10: International Classification of Diseases, 10th revision

ICD-10 code category by organ system	Number of admissions (%)
Certain infections and parasitic diseases	5865 (9.4)
Neoplasms & diseases of the blood and blood-forming organs and certain disorders involving the immune mechanism	3115 (5)
Endocrine, nutritional, and metabolic diseases	2625 (4.2)
Mental, behavioral, and neurodevelopmental disorders	2335 (3.7)
Diseases of the nervous system	2160 (3.5)
Diseases of the eye and adnexa, and ear	130 (0.2)
Diseases of the circulatory system	10210 (16.4)
Diseases of the respiratory system	6690 (10.7)
Diseases of the digestive system	6710 (10.7)
Diseases of the skin and subcutaneous tissue	2945 (4.7)
Diseases of the musculoskeletal system and connective tissue	5825 (9.3)
Diseases of the genitourinary system	3095 (5)
Pregnancy, childbirth, and puerperium	2505 (4)
Congenital malformations, deformations, and chromosomal abnormalities	50 (0.08)
Symptoms, signs, and abnormal clinical laboratory findings, not elsewhere classified	2625 4.2)
Injury, poisoning, and certain other consequences of external causes	5020 (8)
Factors influencing health status and contact with health services	525 (0.8)
Specific principal discharge diagnosis	Number of Admissions (%)
Sepsis	3815 (6.1)
Acute COPD exacerbation	1345 (2.2)
Acute kidney injury	1055 (1.7)
Discoid lupus erythematosus	890 (1.4)
Non-ST elevation myocardial infarction	835 (1.3)
Hypertensive heart and CKD with HF	775 (1.2)
Primary osteoarthritis of right knee	740 (1.2)
Hypertensive heart disease with HF	735 (1.2)
Urinary tract infection	680 (1)

## Discussion

This in-depth nationwide analysis identifies specific differences between hospitalized SLE and CLE patients that may lead to differences in hospital outcomes. 

Our data demonstrate that the CLE cohort was older in age, had better hospital outcomes, and a majority were White in ethnicity. In comparison, the SLE cohort had more co-morbidities and the majority were Black in ethnicity. This supports prior studies that have found a higher prevalence of SLE amongst Black patients, particularly Black women, in addition to greater disease severity in comparison to other racial groups [10,11]. In a study analyzing the mortality trend of SLE patients across 46 years, Black patients suffered a higher mortality rate and at a younger age in comparison to their White counterparts [12]. Furthermore, prior studies have identified Black SLE patients as having higher rates of progression toward end-stage renal disease [13]. 

Our finding of the CLE cohort being predominantly White may be due to the under-diagnosis of CLE findings in non-White patients. In an expert opinion piece, McMichael discussed the ongoing challenge for dermatologists in diagnosing skin diseases in patients of color [14]. There is a lack of skin type representation among dermatology training materials, medical textbooks, and web-based medical resources [15,16]. A study conducted by Fenton et al. identified medical students were less accurate in diagnosing dermatologic conditions in more pigmented skin compared to lighter skin tones [17]. These dermatologic healthcare disparities may disproportionately impact minority patients, which can lead to either a missed diagnosis or cause the patient to present later in their disease onset with possible progression to SLE. 

We found the CLE cohort was significantly older in comparison to their SLE counterpart. Because CLE is limited to cutaneous findings, it is more likely that the CLE cohort was admitted for medical comorbidities associated with older age. Older patients have associated increased CV risk, and CV disease is estimated to cause 40% of all deaths in patients 65 years and older [18]. Sepsis was also notably a high burden of disease to older adults, who were more susceptible due to reduced immune response to infection, increased risk of malnutrition, and were more likely to be institutionalized at a care facility [19]. A prior study identified high sepsis-related mortality and high hospital utilization among advanced-age patients [20]. Furthermore, the elderly population was at risk of loneliness and psychological distress from potential social isolation due to the retirement or death of loved ones [21]. A prior study performed a financial analysis in CLE patients with and without depression. The study found that CLE patients with depression had higher hospital utilization, prescribed medications, and overall hospital cost [22]. This was reflected in our data as our CLE cohort was older in age, had increased CV risk factors, and was more frequently admitted for CV disease, sepsis, and mental health disorders compared to the SLE counterpart. 

Patients with CLE on immunosuppressive medications such as methotrexate are also at increased risk of infections due to the medications. CLE can lead to reduced perceived aesthetic appearance, which can be associated with depression and other mental health disorders [22]. Our study emphasizes the importance of close clinical monitoring of CLE patients in the outpatient setting to lower hospital utilization and cost. Among patients with CLE, it is important to have a prompt diagnosis and treatment of infectious disease, routine mental health screening, and appropriate management of CV co-morbidities/risk factors such as hypertension, dyslipidemia, type 2 diabetes mellitus, obesity, and tobacco use.

There are several strengths of our study. First, it used a nationally representative database to evaluate demographic characteristics and hospital outcomes between the two cohorts. The large sample size from the database provides statistical power to our analysis. However, there are some limitations. The NIS data is based on billing codes; therefore, there is a possibility of error due to incorrect input of ICD-10 codes. The NIS data reports data on hospitalization, rather than patients; therefore, an individual patient will be counted multiple times if they have recurrent admissions. NIS does not contain data on laboratory results and the age of disease onset. 

## Conclusions

CLE patients who were hospitalized were older in age, had less co-morbidity burden, and experienced greater CV risk factors compared to their SLE counterparts. Also, the CLE cohort had a greater percentage of White ethnicity and fewer minority patients. Disorders of the circulatory system were the most common reason for hospitalization by organ system among the CLE cohort, while sepsis was the most common principal diagnosis. Our study highlights the importance of the management of co-morbidities (such as CV risk factors) and early detection of infections in the outpatient setting among CLE patients to help improve long-term hospital outcomes and lower hospitalization costs. 
